# Ankrd11 is a chromatin regulator involved in autism that is essential for neural development

**DOI:** 10.1186/2193-1801-4-S1-L28

**Published:** 2015-06-12

**Authors:** Anastassia Voronova, Denis Gallagher, Mark Zander, Gonzalo Cancino, Alexa Bramall, M. P. Krause, C. Abad, M. Tekin, P. M. Neilsen, D. F. Callen, S. W. Scherer, G. M. Keller, D. R. Kaplan, K. Walz, F. D. Miller

**Affiliations:** Hospital for Sick Children, Canada

**Keywords:** Chromatin, stem cells, brain development

Ankrd11 is a potential transcriptional regulator that is implicated in cognitive dysfunction and ASD, but has no known function in the brain. We show that Ankrd11 is expressed in the embryonic cortex, and that when it was knocked-down in murine cortical precursors this caused decreased proliferation, reduced neurogenesis, and aberrant neuronal positioning in developing cortex. Knockdown of Ankrd11 in human forebrain neural precursors phenocopied Ankrd11 knockdown in murine neural precursors. Decreased proliferation of embryonic and adult neural precursors and neuronal mispositioning were also observed in Yoda mice carrying a point mutation in the histone deacetylase (HDAC)-binding domain of Ankrd11. Yoda mice also displayed ASD-like behavioural abnormalities. Consistent with a role for Ankrd11 in histone acetylation, Ankrd11 was associated with chromatin and co-localized with HDAC3. Also, expression and histone acetylation of Ankrd11 target genes were altered in Yoda neural precursors. Finally, the Ankrd11 knockdown-mediated decrease in precursor proliferation was rescued by inhibiting histone acetyltransferase activity or expressing HDAC3. Thus, Ankrd11 is a crucial epigenetic regulator of neural development that regulates histone acetylation and gene expression, thereby providing a likely explanation for its association with cognitive dysfunction and ASD.

